# Quantitative evaluation of individual food intake by insectivorous vespertilionid bats (Chiroptera, Vespertilionidae)

**DOI:** 10.1242/bio.058511

**Published:** 2021-06-07

**Authors:** Marharyta Moiseienko, Anton Vlaschenko

**Affiliations:** 1 Bat Rehabilitation Center of Feldman Ecopark, 62340 Lisne, Kharkiv Region, Ukraine; 2 H.S. Skovoroda Kharkiv National Pedagogical University, Institute of Natural Sciences, Valentynivska St., 2, Kharkiv, 61168, Ukraine; 3 NGO, Ukrainian Independent Ecology Institute, Plekhanivska St., 40/27, 61001 Kharkiv, Ukraine

**Keywords:** Ecosystem services, Insects, Insectivorous bats, Chiroptera, Ukraine

## Abstract

Insectivorous bats provide important ecosystem services, especially by suppressing and controlling the insects’ biomass. To empirically quantify the number of insects consumed by European vespertilionid bats per night, we estimated their ratio of dry mass of feces to mass of consumed insects. This study combines the results of feeding in captivity and the data obtained in field surveys; dry mass of feces was measured in both cases. In captivity, we analyzed the effect of species, age and sex of bats, species of insects consumed and the mass of food portion on the dry mass of feces. Using coefficients of the regression model, we estimated the number of insects consumed by free-ranging bats based on dry mass of their feces. According to our estimates, on average, one individual of one of the largest European bat species, *Nyctalus* *noctula,* consumes 2.2 ***g*** (ranging from 0.5 to 8.2 ***g***) of insects per one feeding night, while the smallest European bats of genus *Pipistrellus* consume 0.4 ***g*** (ranging from 0.1 to 1.3 ***g***), further confirming the importance of insectivorous bats for ecosystem services. This publication offers the novel method for the estimation of insects’ biomass consumed by bats.

## Abstract (precis)

**Summary:** This study applies a combination of experiment in captivity and field survey that can be used as an easy methodological approach to estimate insect biomass consumed by bats.

## INTRODUCTION

Insectivorous bats play a key role in suppressing insect populations, including crops’ and monoculture tree plantations’ pests. Published data suggest that insectivorous bats belong to the main controllers of arthropod pests (Boyles et al., 2011; Kunz et al., 2011; Russo et al., 2018) suppressing insects due to their nightly foraging activities. At the same time, we are still far from an exact estimation of the volume of ecosystem services bats provide, not only globally but even regionally (Boyles et al., 2013). One of the main obstacles to such estimations is limited data on the actual number of insects each bat consumes (Boyles et al., 2013).

Several publications provide some estimates of the number of insects consumed by bats, covering a broad geographical, ecological and species range (Simmons, 2005; Kunz et al., 2011; Kasso and Balakrishnan, 2013). Bats control the number of insects thereby greatly contributing to cost-saving in agriculture. We can, as an example, present a monetary equivalence estimation of ecological services provided by bats using data available for North America and South Africa. The pest control provided by *Tadarida* *brasiliensis* saves cotton farmers in south-central Texas US $6 million per year (Cleveland et al., 2006), and such pest control services are worth roughly US $22.9 billion annually across North America as a whole (Boyles et al., 2011). In South Africa, bats annually save between 0.53% and 1.29% of the total volume of macadamia nut production and reduce damage cost by 9 to 23% (US$613 ha^−1^) (Taylor et al., 2017). On farmland, bats consume adult insects thereby also reducing the number of laid eggs (Boyles et al., 2013; Russo et al., 2018; Maine and Boyles, 2015). The first estimation of the number of insects consumed by bats in Europe was recently performed by Aizpurua and Alberdi (2020), who calculated that six cave- and forest-dwelling species of European bats consume 63.3±13.9 metric tons of insects daily over the whole territory of Europe. However their estimations based on bat population density only for one region of Europe, and according to that the all extrapolation is far from accurate (Aizpurua and Alberdi, 2020). Birds and bats are major controllers of insect pests in fields, in tropical or neotropical forests and in agricultural landscapes (Maas et al., 2015; Kimberly et al., 2008; Kalka et al., 2008; Morrison and Lindell, 2012). The above studies demonstrate the indispensability of insectivorous bats in agriculture and in wild ecosystems in general, however, there are still blind spots in our understanding of interactions between bats and their prey.

Recent studies (Hallmann et al., 2017; Sánchez-Bayo and Wyckhuys, 2019) show that insect biomass and biodiversity are decreasing regionally and globally. Populations of large insects, such as Lepidoptera and Coleoptera, are particularly affected, whereas the population of Diptera is growing, especially in urban ecosystems (Sánchez-Bayo and Wyckhuys, 2019). However, several authors criticize the methodology of these studies and suspect probable overestimation of the results (Thomas et al., 2019; Didham et al., 2020), while other researchers suggest that populations of insects are indeed declining, which altogether indicates that this topic needs further research to obtain accurate estimates. Anyway, changes in the numbers of insects may affect bat reproduction. Bats are more selective in their diet during the breeding season (Anthony and Kunz, 1977) and their activity largely depends on insect abundance (Heim et al., 2017; Maine and Boyles, 2015). Investigation of prey–predator interactions between bats and insects can reveal new invisible threats in addition to the known ones (Frick et al., 2019; Owens et al., 2020; Didham et al., 2020). Decreasing populations of insects and extinction of some insect species may lead to changes in the status of some bat species within the existing ecological niches, in particular, within specialized niches. Some bat species, whose diet is made up of insects that belong to species suffering rapid declines, may face the threat of population decline despite all strategies aimed at their protection. The most significant threat this would pose onto populations of large insectivorous bats (e.g. *Nyctalus* *noctula*). Their diet consists of large prey (Beck, 1995), and research findings suggest that they will not be able to make up the energy costs by eating small insects (Wetzler and Boyles, 2018). At the same time, small bats that predate on Diptera or small Lepidoptera may benefit from the situation. One of the reasons for fast distribution of *Pipistrellus* *kuhlii* (Hukov et al., 2020) in cities can be the availability of small insects. To be able to predict such changes and threats, we need to estimate the mass of insects that different bat species need to consume to survive, and the method used for such estimation must be simple, applicable and ethically acceptable.

Therefore, we aimed to evaluate the effect of different factors on the digestion level (assessed by the dry mass of feces) in captive bats and, based on these results, to estimate the amounts of insects consumed by bats in the wild. In captive settings, we collected feces from animals of three bat species (*N.* *noctula, Eptesicus serotinus, P. kuhlii*) after feeding each bat a portion of insects of a known weight. In addition, we used mist nets to capture four common, widespread species of free-ranging bats (*N. noctula, P. kuhlii, Pipistrellus nathusii,* and *Pipistrellus pygmaeus*), and collected their feces over a period of at least 12 h to include all intestinal contents formed from the previous night foraging. Based on this data, we would estimate the volume of insects eaten during one feeding session. One of the main purposes of this study was to test the applicability of this approach to estimate the number of insects eaten by bats, and to investigate possible limitations and biases of this method.

## RESULTS

### Rates calculated in in-captivity experiment

**Fig. 1.**
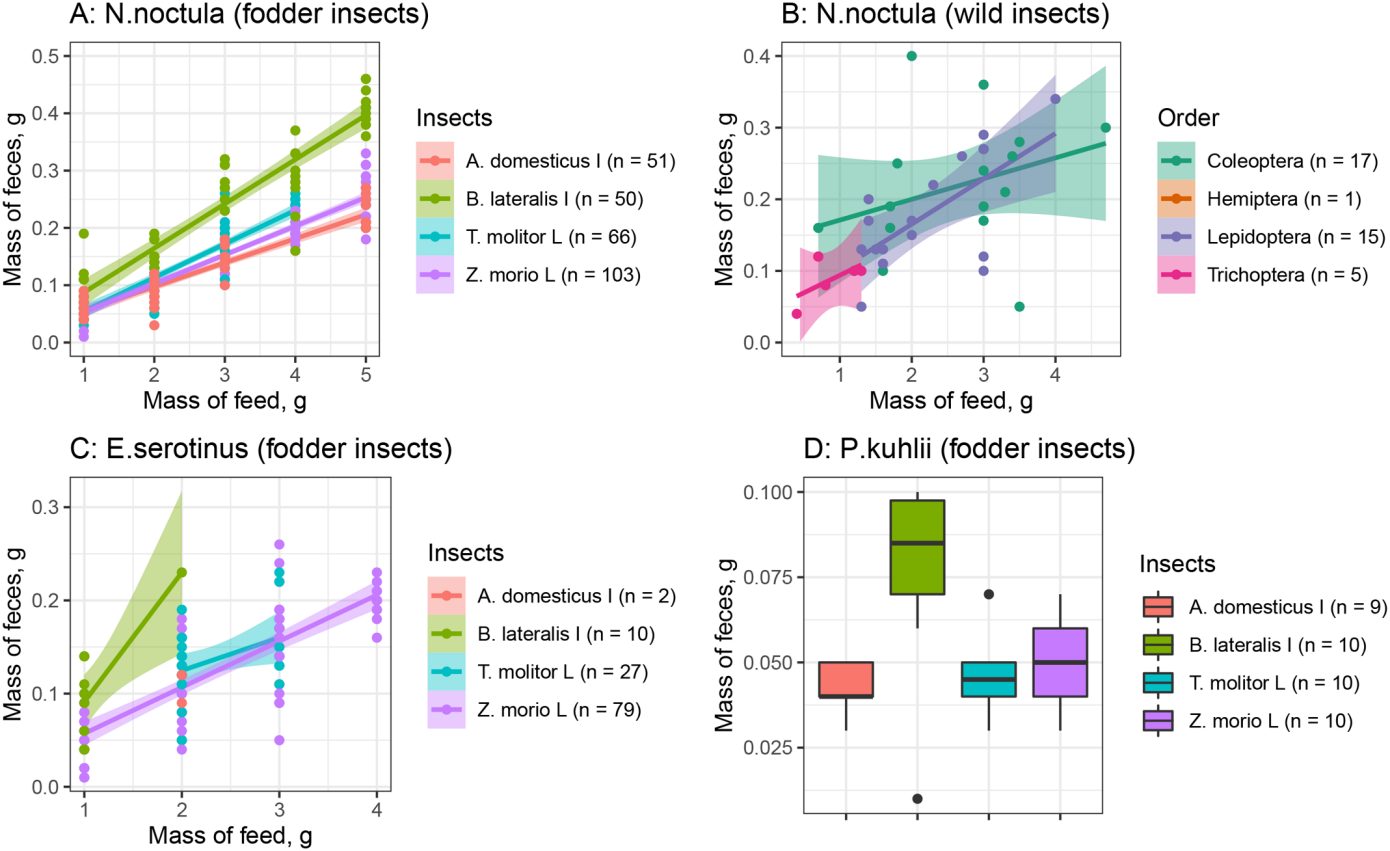
**Visualization of linear models for the mass of feces of bats versus the mass of feed provided.** Mass of feces of *N. noctula* and *E. serotinus* presented on charts A (fodder insects), B (wild insects) and C. Lines are trend lines, shaded areas are 95% confidence intervals, points represent individuals used in the experiment. Dry mass of *P. kuhlii* (D) feces after consumption of 1 ***g*** of feed containing one of the four insect species (lines are median, box: 25/75 percentile, dot: outlier). Stages of insect metamorphosis are marked as: L, larva and I, imago; the fodder insects belong to such orders *Z. morio*, *T. molitor* - Coleoptera, *A. domesticus* - Orthoptera, *B. lateralis,* Blattodea.

Multiple regression analysis of linear model (LM) (1) for mass of feces (Table 1) showed that the mass of feces (from the same mass of fodder) did not differ significantly between *E. serotinus* and *P. kuhlii* (*P*>0.1) as well as between *E. serotinus* and *N. noctula* bats (*P*>0.1). There were also no significant differences between males and females as well as between adult and subadult bats (*P*>0.1). The interaction species×sex×age had no significant effect (*P*>0.1). Coefficients of food taxon (*Blatta* *lateralis* as an intercept) were significantly higher for Coleoptera (*P*<0.01); no significant differences were found in other comparisons (*P*>0.1). The mass of the food portion had a significant effect (*P*<0.01) on the mass of feces. Coefficients of the mass of food portion significantly decreased when food taxon changed to any other species of fodder insects (*P*<0.01): *Zophobas* *morio*, *Tenebrio* *molitor*, or *Acheta* *domesticus*. At the same time, coefficient of the mass of food portion for *B. lateralis* did not significantly differ from the respective coefficient for wild insects Lepidoptera (*P*<0.1), Coleoptera (*P*>0.1) and Trichoptera (*P*>0.1). During the warm season the average mass of feces was lower compared with the cold season (*P*<0.01). The interactions food taxon×season and mass of food portion×food taxon×season had no significant effect. Visual representation of LM (1) is shown in Fig. 1.

**Table 1.**
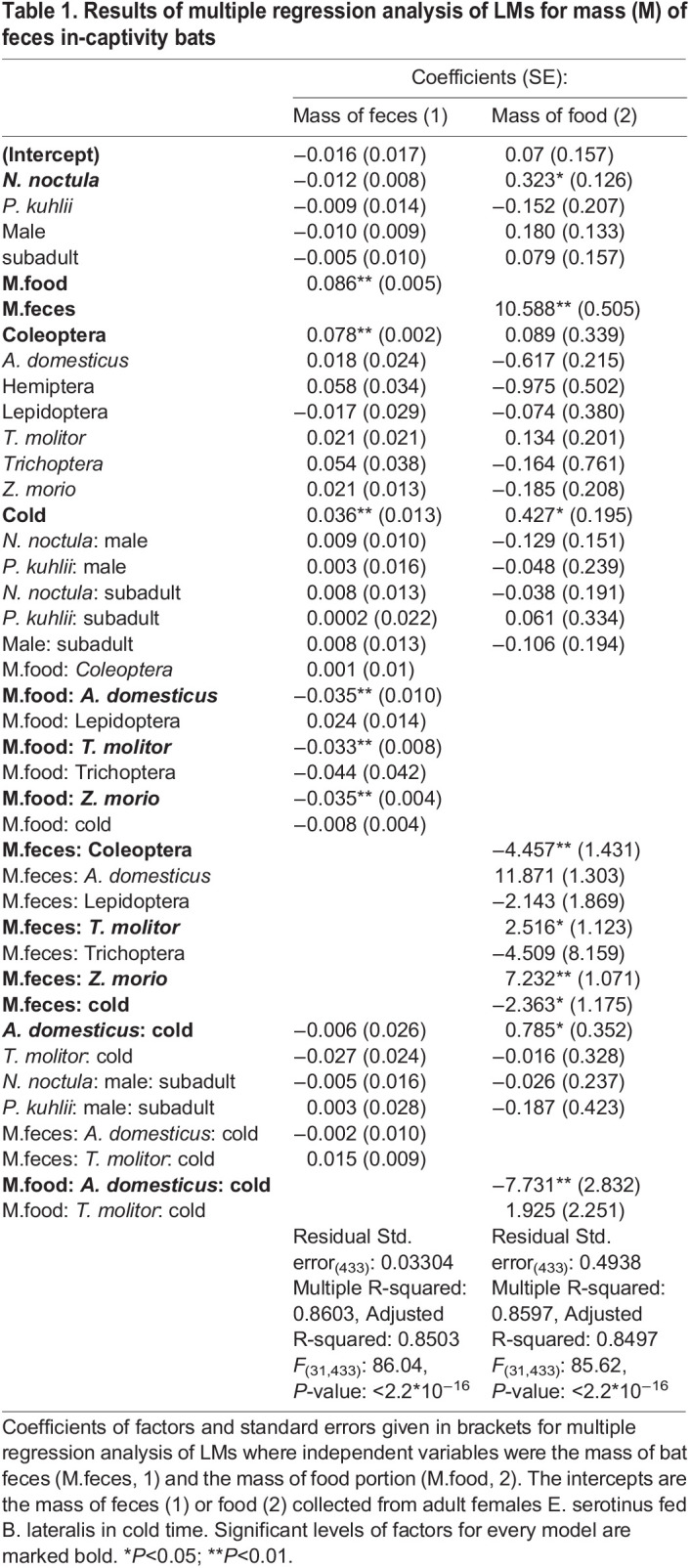
Results of multiple regression analysis of LMs for mass (M) of feces in-captivity bats

Finally, we found no significant effects of bat species, sex, age and season, whereas food taxon of insects used as a food significantly affected the mass of feces. Consumption of 1 ***g*** of insects resulted in formation of 0.086 ***g*** of feces for *B. lateralis* or Coleoptera insects, and 0.04–0.06 ***g*** of feces for other fodder taxons (Table 1, M.food: food taxon interaction).

The inverse coefficients in the LM (2) were similar, but in some cases the significance levels for differences were higher (Table 1).

### Estimated mass of insects consumed by local bat populations

The results of multiple regression analysis (Table 2) showed significant differences in the mass of feces between free-ranging *N. noctula* and *Pipistrellus* (*P*<0.05). There were no significant differences in sex, age, time of catching in both parts of night and interactions of these factors between the species (*P*>0.1).

**Table 2.**
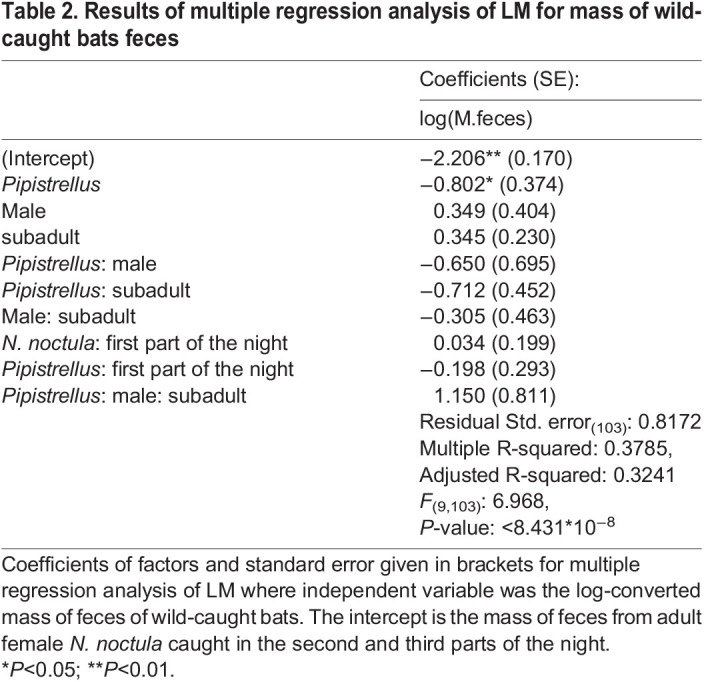
Results of multiple regression analysis of LM for mass of wild-caught bats feces

**Fig. 2.**
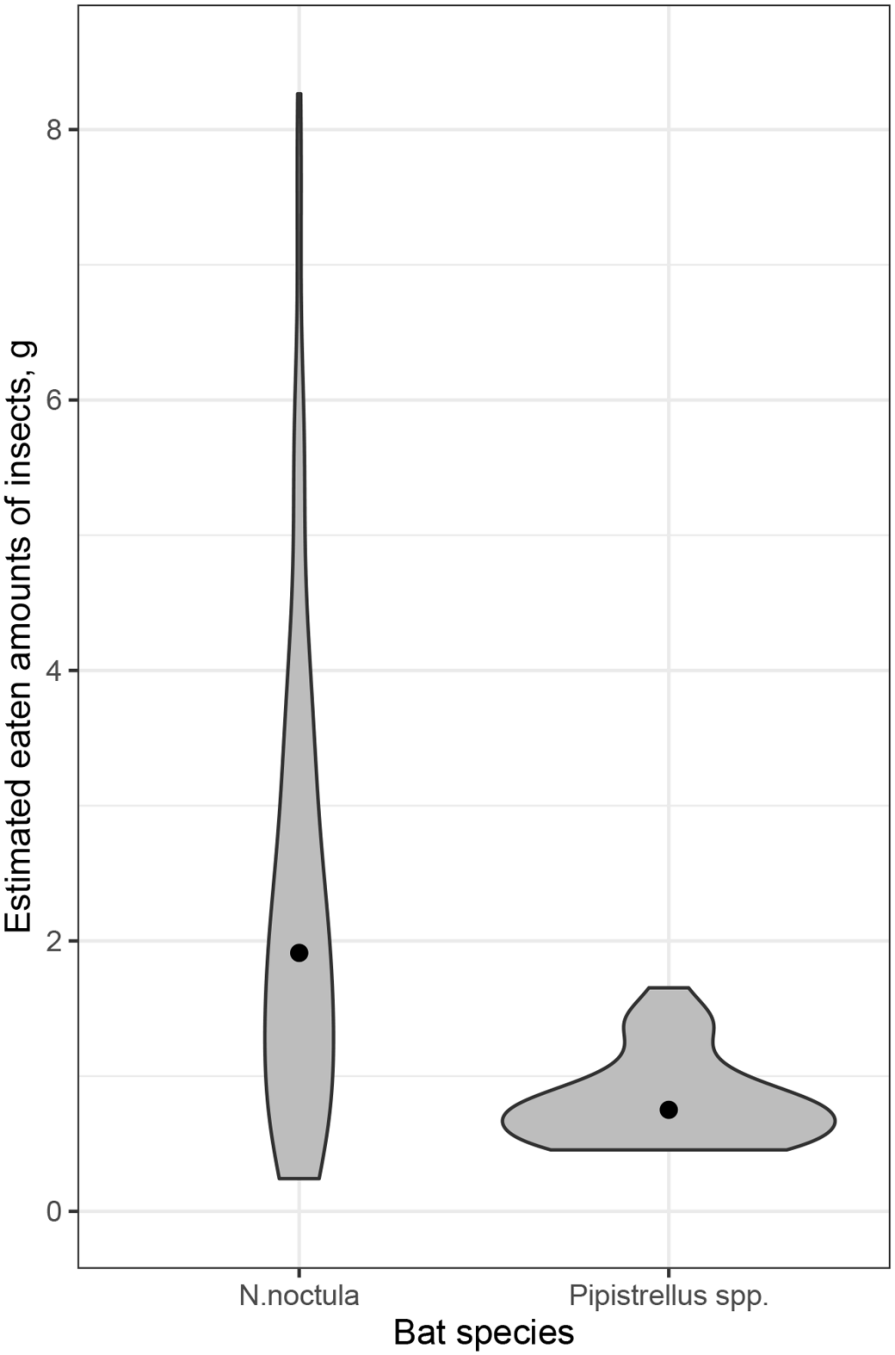
Violin plots for estimated amount of food consumed by free-ranging *N.noctula* (*n*=95) and Pipistrellus spp.* (*P. nathusii, P. pygmaeus, P. kuhlii*; *n*=33) during one feeding session. Black dots mark median values and grey-colored areas mark the distribution of estimated amounts of food. *The data for the mass of feces from the three *Pipistrellus* species were combined.

We used LM (2) coefficients to predict the mass of insects consumed by free-ranging bats. According to the results, one *N. noctula* bat eats 2.2 ***g*** (range: 0.5–8.2 ***g***) and *Pipistrellus* bat eats 0.4 ***g*** (range: 0.1–1.3 ***g***) per night (Fig. 2).

## DISCUSSION

In our study, we combined the results of experiments carried out in captivity and in field settings to evaluate the individual food intake by insectivorous bats. Our findings add important details and information on quantification of the bat feeding ecology. We also suggest that the novel method proposed here may be used in future research to calculate the amounts of insects consumed by bats to investigate prey-predator interactions in field studies.

### Results of in-captivity bat feeding

We did not observe any significant differences in the ratio of the mass of feces and the level of food digestion in bat species studied. However, such pattern was observed in other studies; Kovtun and Zhukova, 1988 showing positive correlations between the size of a bat and the level of digestion and Alekseeva et al., 1980 and Pervushina et al., 2010 showing negative correlation, but the impact of the bat species has not been verified statistically.

We did not find any effect of either sex or age on the mass of feces was found for in any of the bat species studied here, either in captivity or in the wild. However, it was shown that during the breeding period females consume more food, which corresponds to their higher energy demands (e.g. Kunz, 1974; Encarnação and Dietz, 2006). The lack of statistically significant differences in our study might have been due to the fact that pregnant or lactating females and juveniles had been excluded from the captivity experiment.

We observed an effect of the season on the mass of feces. During warm periods of the year, feces were heavier than during colder periods. In other words, digestion level during hibernation is higher than in summertime (under conditions of artificial feeding in the BRC-FE). Possible explanation might be that bats are able to receive more energy from lower amounts of insects during winter or early spring arousal.

As we had expected, there was a statistically significant effect of insect species used as a food on the mass of feces. Previously, it was shown that insects of different species and stages of development were digested at different levels depending on the amount of chitin in insect bodies (Staliński, 1994). Among the four species of fodder insects analyzed, the lowest masses of feces and the highest level of digestibility were observed after consumption of *A. domesticus* imagos and *Z. morio* and *T. molitor* larvae. In contrast, consumption of *B. lateralis* imagos resulted in formation of a greater mass of feces, and this mass was similar to those obtained after consumption of wild insects (Fig. 1 and Table 1), therefore the ratio obtained for *B. lateralis* was used to estimate the mass of food eaten by free-ranging bats. Previously, the difference in the digestion level of fodder and wild insects was shown for *N. noctula*’s feces: the mass of feces was twice higher after consumption of wild insect imagos compared with *T. molitor* larvae (Alekseeva et al., 1980). However, Alekseeva et al., (1980) do not mention what the orders of ‘wild insect imagos’ were used. Our analysis showed no differences between the orders of wild insects (Table 1). However, this might be due to a small sample size. In summary of this section, we suggest that *B. lateralis* should be used in experiments with other bat species (similar in size to *N*. *noctula*) in captivity to estimate the insect consumption by free-ranging bats.

### Calculating amounts of insects consumed by free-ranging bats in the wild

In contrast to the results of other studies (Kunz et al., 1995; Encarnação and Dietz, 2006; Anthony and Kunz, 1977) and the general idea of a direct correlation between the bat body mass and the insect consumption rate (Boyles et al., 2011; Kunz et al., 2011; Russo et al., 2018), our extrapolation of mass of feces to mass of wild insects in nature looks scarce (Fig. 2).

According to our calculations, the mean consumption rate for *N. noctula* was 7.8% of the body mass (28 ***g*** was used as an average body mass for calculations) reaching up to 29.3% of the body mass (maximum), and the mean consumption rate for *Pipistrellus* bats was 6.6% (6 ***g*** was used as an average body mass for calculations) reaching up to 33.3% (maximum). Therefore, our estimates present the amount of food that actually reached the bat's stomach, excluding the mass of non-eaten insect parts (i.e. chitin, wings, legs, etc.) that bats left out, and, according to the literature (Coutts et al., 1973) the percentage of these parts might be up to 20–50% from the initial mass of insects. The mass of insect parts was not included in our case, which probably made our estimates underestimate. Moreover, we did not take into account the effect of drinking water on the level of food digestion. In our in-captivity experiment we did not provide bats with any water on the day of feeding, but in the wild they consume a lot of water. On the other hand, it has been also shown the level of digestion is higher in active (flying) individuals compared with inactive ones (Alekseeva et al., 1980; Kovtun and Zhukova, 1988). Taking into account all of these limitations and assumptions, we assume that even if our rate is underestimated, the actual value should not exceed by more than 10 to 20%. With this correction, the mean mass of insects consumed (Fig. 2) may be around 2.5 ***g*** (9% of body mass) for *N. noctula* and at around 0.5 ***g*** (12% of body mass) for *Pipistrellus spp*. In any case, these slightly higher amounts would not significantly change the number of insects consumed by one bat. However, these amounts still appear to be insufficient for periods of high energy requirements, such as during pregnancy, lactation, and spermatogenesis, according to estimates published by other authors (Encarnação and Dietz, 2006; Anthony and Kunz, 1977). Due to high food passage rate, the bat stomach fills and empties overnight. They do not carry the whole mass of eaten insects in their digestive tract during the whole night. Indeed, our results showed that each individual caught in the wild at some point of time during the night carries in its digestive tract only some of the insects eaten. Summarizing the extrapolation from the field, we can say that our findings are possibly an underestimation.

At the same time, our results are consistent with some published studies, where the daily food intake did not exceed 33% (McLean and Speakman, 1999; Kunz, 1974; Anthony and Kunz, 1977) of the bat body mass. For example, the average dry food consumption of a non-reproductive individual of *Plecotus auritus* species was 1.8 ***g***, and a lactating female consumed 2 ***g*** (McLean and Speakman, 1999), but this estimate was provided on dry mass of insect and did not include water mass in food. In the study of *Myotis velifer* feeding ecology (Kunz, 1974), males had a lower consumption rate in May (0.47 ***g***, or 4% of the body mass) and females ingested the largest amount of food (2.7 ***g***, or 23% of their body mass) during lactation at the end of July. Anthony and Kunz's (1977), using similar field survey methods, showed that pregnant and lactating females of *Myotis lucifugus* species consumed on average 2.5 ***g*** (32% of the body mass) and 3.7 ***g*** (46% of the body mass) of insects, while juveniles of this species ingested 1.8 ***g*** (22.5% of the body mass). They assumed that *M. lucifugus* had two foraging periods per night, whereas here we have taken only one period, and for their calculation of the amount of food consumed they used the value of the body weight loss assessed for bat groups. In captivity, the maximum estimated food consumption was 40% from the body mass (Kovtun and Zhukova, 1988). Further, our unpublished data on feeding captive bats at the BRC-FE showed that in some cases, the mass of food consumed by a single *N. noctula* individual could reach up to 32–50% of its body mass. In addition, maximally full stomach seems to be associated with a decreased flying activity in bats (as was evident from our observations made at the BRC-FE with subadult individuals that were not willing to fly after feeding, unpublished data) that could lead to a less successful foraging.

On the other hand, according to Encarnação and Dietz (2006), one *Myotis daubentonii* female eats between 4.3 and 8 ***g*** of insects (50–84% of the body mass) per a day during pregnancy and between 2.7 and 4.9 ***g*** (28–51% of the body mass) in the post-lactation period. Males in their study consumed 1.9 to 3.6 ***g*** of insects (32–43% of the body mass) during late spring and 4.4–8 ***g*** (53–96% of the body mass) during the period of intensive spermatogenesis. The advantage of their study design was that they tracked the time spent foraging, while the limitation of study was that they calculated the amount of the insects consumed based on the capture success rate, and not the actual change in the bat body weight. Kunz et al. (1995) reported that the amount of insects consumed daily by one *T. brasiliensis* female was between 39 and 73% of its body mass (4.4–8.3 ***g*** of insects per 11.5 ***g*** of the bat body mass) during early and mid-lactation, while in some cases this percentage even reached 90 to 100% (Kunz and Stern, 1995). Pregnant females of *Myotis lucifugus* consume 5.5 ***g*** of insects per day (61% of the bat body mass) and lactating females of this species consume 6.7 ***g*** (84% of the bat’s body mass) (Kurta et al., 1989). These studies show a high percentage (39–96%) of food consumption relative to the body mass of an animal during the active reproductive period. For non-reproductive bats, the consumption rate assessed in published studies was lower than that (43–51%), but it was still higher than the rate seen in our results. Also, these studies (Encarnação and Dietz, 2006; Kunz and Stern, 1995) investigated only bats with an active reproductive status, and their results were based on the metabolic rate in captivity that may differ for bats in the wild. In addition, in the above studies the mass of whole insects was used in calculations. But, as we described in the Materials and Methods, bats leave out some parts of insects’ bodies.

In conclusion, based on literature analysis and the results of this study, we see that the estimated mass of insects consumed by bats is still far for an exact value, since the impact of such factors as animal's sex, age, reproductive status and body size have not been adequately addressed yet. Therefore, more research needs to be done both in captivity and in the wild. More accurate estimates can be obtained in the future, when using a combination of the method proposed here with published energy demand assessment methods. To the best of our knowledge, the method applied in this study is the first quantitative estimation of bats' food intake based on the combination of the results of in-captivity bat feeding and the data of a field survey. Taking into account the innovative nature of our work, we have uploaded all raw datasets as Supplementary Materials so that other bat research groups could apply (to validate or modify) our methodological approach. The coefficients (Table 1) were sufficiently verified and can therefore be used for future research. Possibly, a similar conversion rate could be developed for tropical frugivorous bats.

The analysis of literature on bat feeding ecology and digestion performed for this study, has further supported the idea that bats remain the understudied group of vertebrates. However, bats provide a useful and simple model for nutrition and digestion studies to facilitate additional research. With this study, we tried to add valuable information about bats ecosystem services, but a number of questions about bats feeding behavior and distribution of populations still need to be answered. Further research using improved methods should be able to provide a more profound understanding of the importance of bats in ecosystems.

## MATERIALS AND METHODS

### General study design

Our study was divided into two experiments. Experiment 1 consisted of feeding captive bats pre-weighed insects and collecting their feces for analysis. Experiment 2 consisted of collecting feces from free-ranging bats for analysis. The established relation between the mass of food consumed and the mass of dry feces in captivity, was then extrapolated onto free-ranging animals to estimate the average amount of insects consumed by bats in the wild based on their dry mass of feces.

### Experiment 1. Feeding bats in captivity

The in-captivity experiment was carried out at the Bat Rehabilitation Centre of Feldman Ecopark (BRC-FE) in 2018-2019. This experiment was approved by the Ethics Committee of V.N. Karazin Kharkiv National University (Decision #02/2018). During the experiment, no bats were sick or critically emaciated. Three bat species were used in the experiment: *N.* *noctula* (Schreber, 1774), *Eptesicus serotinus* (Schreber, 1774), and *P.* *kuhlii* (Kuhl, 1817). Study groups included randomly selected individuals excluding pregnant and lactating females. In total, 199 animals were used in this experiment; details about species, sex and age of used bats are in Table S1. No bat was used for this experiment during two days in a row.

Bats were fed larvae (*Z.* *morio* and *T.* *molitor*) and imagos (*B.* *lateralis* and *A.* *domesticus*). Each feeding session included only one type of food. Light-trapped wild insects of four orders were added to the diet of captive bats; see below for further details. Some parts of wild-caught insects (e.g. chitin, wings, legs and elytrons (for Coleoptera) could be left behind by bats. These remains were weighed, and this weight was subtracted from the total mass of the food.

Bats were weighed before and after feeding, on an electronic scale (TANITA 1479V) (accurate to 0.1 ***g***). The records included: date, mass of food portion, body mass before and after feeding, sex, age category (adult or subadult; in this study the term ‘subadult’ is equal to previously used term ‘this-year-born’ (Kravchenko et al., 2017; Hukov et al., 2020) and number of individual rings. On the day of feeding, bats were deprived of water and kept under conditions of limited physical activity after feeding (were not allowed to fly). After feeding each bat was kept in an individual small fabric holding bag for at least 12 h (up to 20 h) at 20–25°C regardless of the season. According to previous studies (Kovtun and Zhukova, 1988; Staliński, 1994), this time (8–12 h) should have been enough to eliminate at least 90% of the contents of the digestive tract of bats.

After this period, feces were collected into individual paper envelopes. The envelopes with feces were dried in a thermostat (drying cabinet SSh-2V-151) at 40°C for 6 h. This temperature was chosen to preserve DNA for future genetic analyses of samples from free-ranging bats. Dried feces were weighed on an electronic jewellery scale with accuracy to 0.01 ***g***.

In total, we collected 465 samples (308 *N. noctula,* 118 *E. serotinus*, 39 *P. kuhlii*) of dried bat feces during Experiment 1. The total amount of fodder insects fed to bats was 1060 ***g***. All raw data are summarized in Table S1 and Table S2 provided as electronic supplementary material.

### Insect light trapping

A light trap was placed at a height of 3 m in the Regional Landscape Park ‘Feldman Ecopark’ (50°06′04′′N 36°17′20′′E), in the oak forest, where it remained over the period from 30 May to 1 September, 2019. The light trap worked at night to capture nocturnal flying insects. The trapped insects belonged to orders Coleoptera, Lepidoptera, Hemiptera, and Trichoptera. The total number of samples of wild insects was 39 and the total weight of all samples was 83 ***g***.

### Experiment 2. Bat capturing in the field

During summertime (between 1 July and 10 August in the years 2018 and 2019), bats were caught in ultra-thin mist nets at several woodland locations in Kharkiv and Kyiv Regions, according to the published protocol of bat catching (Vlaschenko et al., 2016). Information about animals and places where bats were caught is shown in Table S2. Immediately after each bat was taken from the mist net, it was ringed, weighed, sexed, aged and put into a clean fabric holding bag. The exact time of catching was recorded. After at least 12 h (up to 18 h) in the holding bag, fecal pellets were collected into individual paper envelopes, and bats were released to the wild after sunset. Fecal samples were then dried and weighed according to the same procedure as during the in-captivity experiment. We collected 128 samples of feces from 128 free-ranging bats: 95 samples from *N. noctula* and 33 from three species of genus *Pipistrellus* (1 *P. kuhlii*, 22 *P. nathusii*, 10 *P. pygmaeus*).

### Data analysis

Visualization and statistical analysis were performed using R software (version 4.0.0., R Core Team 2020), charts were created using ggplot2 package. Linear models (LMs) were built via lm() function and their values were calculated using summary(). All results were considered significant at *P*≤0.05.

We built LM (1) for mass of feces, where the mass of food portion, sex, age, species of bats, insect species (food taxon) used for feeding, season [cold (hibernation time) / warm] and interactions of factors (species×sex×age of bats and mass of feces×food taxon×year season) were included as independent variables. We used multiple regression analysis to assess the relationship between the independent variables and the mass of feces. The inverse LM (2) was built to calculate the potential amount of insect intake based on the known mass of feces from free-ranging bats (*N. noctula* and genus *Pipistrellus*).

We analyzed the effect of sex, age, genus of bats and time of catching (1–3 or 4–8 h after sunset) on mass of feces (with log conversion) from free-ranging *N. noctula* and genus *Pipistrellus* by building a LM followed by multiple regression analysis. We took into account the high food passage rate through the digestive tract in bats (Kovtun and Zhukova, 1988; Staliński, 1994) and time of most active foraging (the latest studies for *N. noctula*, Roeleke et al., 2018, 2020) and evaluated the difference between the first three night hours (the first part of a night) versus other night hours (the second part of a night). No significant difference was found (see Results). So we used data for all collected feces from bats caught during the night for data analysis. We calculated median, minimal and maximum amounts of food eaten by one bat per night. For extrapolation of the results, the coefficient obtained for *B. lateralis* was used, because it was the most approximate coefficient among fodder insects that did not differ significantly to wild insect species (see Results, Table 1).

The amount of food consumed by *E. serotinus* was not estimated, because the number of wild-caught individuals of this species was too low.

## Supplementary Material

Supplementary information
