# Supplementary material for: Quantitative evaluation of individual food intake by insectivorous vespertilionid bats (Chiroptera, Vespertilionidae)
Source: Biol Open. 2021 Jun 7;10(6):bio058511. doi: 10.1242/bio.058511 (PMC8214420; doi:10.1242/bio.058511)
Supplement: Supplementary information [file biolopen-10-058511-s1.pdf]

Table S1

Click here to download Table S1

Table S2

Click here to download Table S2

Biology Open • Supplementary information
